# The Role of Dermal Fibroblasts in Nevoid Basal Cell Carcinoma Syndrome Patients: An Overview

**DOI:** 10.3390/ijms21030720

**Published:** 2020-01-22

**Authors:** Barbara Bellei, Silvia Caputo, Anna Carbone, Vitaliano Silipo, Federica Papaccio, Mauro Picardo, Laura Eibenschutz

**Affiliations:** 1 Laboratory of Cutaneous Physiopathology and Integrated Center of Metabolomics Research, San Gallicano Dermatologic Institute, IRCCS, 00100 Rome, Italy; silvia.caputo@ifo.gov.it (S.C.); federica.papaccio@ifo.gov.it (F.P.); mauro.picardo@ifo.gov.it (M.P.); 2 Oncologic and Preventative Dermatology, San Gallicano Dermatological Institute, IRCCS, 00100 Rome, Italy; anna.carbone@ifo.gov.it (A.C.); vitaliano.silipo@ifo.gov.it (V.S.); laura.eibenschutz@ifo.gov.it (L.E.)

**Keywords:** basal cell carcinoma, skin, nevoid basal cell carcinoma syndrome, Gorlin syndrome, hedgehog pathway

## Abstract

Nevoid basal cell carcinoma syndrome (NBCCS), also named Gorlin syndrome, is a rare multisystem genetic disorder characterized by marked predisposition to basal cell carcinomas (BCCs), childhood medulloblastomas, maxillary keratocysts, celebral calcifications, in addition to various skeletal and soft tissue developmental abnormalities. Mutations in the tumor suppressor gene *PATCHED1* (*PTCH1*) have been found to be associated in the majority of NBCCS cases. *PATCH1* somatic mutations and loss of heterozygosity are also very frequent in sporadic BCCs. Unlike non-syndromic patients, NBCCS patients develop multiple BCCs in sun-protected skin area starting from early adulthood. Recent studies suggest that dermo/epidermal interaction could be implicated in BCC predisposition. According to this idea, NBCCS fibroblasts, sharing with keratinocytes the same *PTCH1* germline mutation and consequent constitutive activation of the Hh pathway, display features of carcinoma-associated fibroblasts (CAF). This phenotypic traits include the overexpression of growth factors, specific microRNAs profile, modification of extracellular matrix and basement membrane composition, increased cytokines and pro-angiogenic factors secretion, and a complex alteration of the Wnt/β-catenin pathway. Here, we review studies about the involvement of dermal fibroblasts in BCC predisposition of Gorlin syndrome patients. Further, we matched the emerged NBCCS fibroblast profile to those of CAF to compare the impact of cell autonomous “pre-activated state” due to *PTCH1* mutations to those of skin tumor stroma.

## 1. Introduction

Nevoid basal cell carcinoma syndrome (NBCCS OMIM #109400), also known as Gorlin–Goltz syndrome or Gorlin syndrome (GS), is a rare genetic alteration with an estimated prevalence of around 1 in 100,000 on average [1,2] and of 1 in 256,000 in Italy [3]. It is most frequently produced by a mutation in the patched homologue 1 (*PTCH1*) tumor suppressor gene, a component of the Hedgehog (Hh) pathway, and it is inherited in an autosomal dominant manner, though about 20–30% of cases present a de novo pathogenic variant. This syndrome shows a high penetrance and variable expressiveness. It is characterized by jaw keratocysts, palmar and/or plantar pits and calcification of celebral falx, malformations of the ribs, macrocephaly, generalized overgrowth and a predisposition to tumors, specifically multiple basocellular carcinomas, medulloblastoma, meningioma and benign ovarian cysts and cardiac fibromas [4,5,6,7,8,9,10,11,12]. Recently, the model of patient-derived induced pluripotent stem cells (iPSC) clarified some of the molecular mechanisms underlying skeletal abnormalities [13]. Clinical diagnosis of Gorlin syndrome requires either two major criteria (multiple basal cell carcinomas (BCCs) (≥5 in a lifetime) or a BCC before 30 years, lamellar calcification of the falx cerebri, jaw keratocysts, palmar or plantar pits, first-degree relative with NBCCS) or one major and two minor ones (lympho-mesenteric or pleural cysts, macrocephaly, cleft lip/palate, vertebral/rib anomalies, preaxial or postaxial polydactyly, ovarian/cardiac fibromas, medulloblastoma, ocular anomalies). Clinical features of NBCCS arise in the first, second, and third decade [2,9]. Although patients with NBCCS can often be diagnosed on the basis of clinical data, genetic tests are useful in many situations. Genetic analysis can be performed to confirm the diagnosis in patients lacking sufficient clinical diagnostic criteria and for prenatal and pre-implantation diagnostic purposes if a defined mutation has been identified in a proband family member. Because patients with NBCCS are predisposed to neoplastic disease, patients who are at risk for the syndrome are encouraged to undergo regular surveillance. 

### 1.1. Clinical and Histopathological Features of Nevoid Basal Cell Carcinoma Syndrome-Basal Cell Carcinomas (NBCCS-BCCs)

Life expectancy in NBCCS patients is not significantly different from the general population [2]. Early diagnosis is important due to complications of NBCCS (most notably medulloblastoma) during childhood and susceptibility to multiple neoplasms in early age. The patients affected by Gorlin syndrome often show frequent skin lesions including multiple nevi, epidermal sebaceous and dermoid cysts, small palmo-plantar depression (pits), hair skin pathes, and numerous basocellular carcinomas (BCCs) distributed randomly over the entire face or body including sun-unexposed area [14,15]. The risk of developing a second BCC is 10 times the risk of unaffected individuals and the number of BCCs may vary from few to thousands [16]. The treatment of choice for multiple BCCs is surgical eradication. Consequently, the aesthetic outcome of surgical treatment of multiple skin tumors represents one of the major problem for these patients. However, depending on BCCs location, number and size, medical therapies used for sporadic BCC can be considered. Combined approaches including surgery supplemented by cryotherapy, laser, photodynamic therapy or topical treatments also are used to preserve normal tissue and prevent disfigurement. Radiation therapy must be avoided as it can cause disease relapse and invasion of BCC years later [17]. Interferon injected directly into tumor lesion demonstrated complete resolution of BCC in some patients [17]. Use of oral retinoids has also been suggested [18]. Recently, several small molecules inhibitors of the Hh pathway, impeding ligand-mediated signal transmission, demonstrated efficacy in the clinical practice. Among these, oral administration of Vismodegib and Sonidegib reduce the development of BCC, rendering the surgical resections less challenging for these patients [19,20,21]. However, side effects related to these systemic therapies limit their long-term use in clinical practice [22]. Although BCCs from syndromic individuals are histopathologically indistinguishable from sporadic BCCs [17,23], the corresponding molecular phenotypes do not fully overlap. NBCCS-BCCs present lower mutation load and UV-induced mutagenesis compared with sporadic BCCs, suggesting that strict sun protection is effective in this genetically predisposed population [24]. Reduced mutational load could explain the relatively indolent clinical course NBCCS-BCCs and the responsiveness to target therapy. In fact, it has been observed that NBCCS patients lack intrinsic resistance to Hh target therapy, such as Vismodegib and Sonidegib [25,26]. However, it is unclear if the scarcity of drug resistance to Vismodegib in Gorlin syndrome is an intrinsic disease trait or if it refers to the fact that in these studies, locally advanced and metastatic sporadic basal cell carcinomas were compared to a more heterogeneous group of NBCCS-BCCs [22,27,28]. As demonstrated for sporadic BCC, exposure to the sun promotes the development of BCCs in patients with NBCCS [29]. The lower frequency of BCCs in the African-American syndromic patients compared with Caucasian populations, presumably due to the protective effect of melanin, confirm that the sun may exacerbate the development of BCCs in NBCCS [30]. However, since NBCCS-BCCs commonly develop also in sun-protected skin area, the role of sun exposure is still debated. Also, data emerging from mouse models are not conclusive since patch knockout mice develop more BCCs than wild mice when exposed to ultraviolet (UV) but about one-third of skin tumors develop in absence of exposure to external genotoxic stress [29] *Ptch1*-deficent mice develop several basaloid hyperproliferation but these cancer precursors progress to nodular and infiltrative BCCs only in X-ray irradiated areas [31]. Also, in vitro studies showed some incongruity. NBCCS fibroblasts display hypersensitivity to UVB, evidenced by accumulation of oxidative damage (8-hydroxydeoxyguanosine and pyrimidine dimers) and reduced cell survival [32,33]. By contrast, Brellier and collaborators reported normal nucleotide excision repair and survival capacity in UVB-treated NBCCS keratinocytes and fibroblasts [34].

### 1.2. Genetic Aspects

The majority of NBCCS cases can be associated with heterozygous germline mutations in *PTCH1* (Chr9q22.32), the human homolog of the Drosophila gene, *patched* [35], and is transmitted in an autosomal dominant way with high penetrance and variable expressivity [36]. Males and females are equally affected. Most germline mutations in NBCCS lead to a premature termination of the PATCHED protein [37,38]. *PTCH1* gene is composed of 23 exons and codifies a transmembrane glycoprotein (PTCH1) composed of 1447 amino acids and 12 domains [7,8,36]. Since the discovery of the *PTCH1* gene as being responsible for Gorlin syndrome in 1996 [4], more than 200 sequence mutations have been reported to date including nonsense, missense, and splicing variants, as well as insertions and deletions [38]. Nearly 20–30% of the syndrome cases present as new mutations [4,6,7,22] and no clustering of mutations has been identified [39]. Nevertheless, missense mutations do not occur with the same frequency as frameshift or nonsense mutations [19]. Data in the literature reported that, despite exhaustive molecular analysis, *PTCH1* mutation could not be determined in some patients, probably due to large deletions or the existence of mutations outside the regions analyzed, such as in introns, intron-exon junctions, or regulatory elements. Other rare causative genes have also been identified in Gorlin syndrome including *PTCH2* and *SUFU* encoding for patched 2 and suppressor of fused homolog proteins, respectively [40,41,42]. In this hereditary setting, the genotype–phenotype correlation is not constantly present: family members sharing the same *PTCH1* germline mutation have a variable phenotype [43]. The missing relationship between the genotype and the phenotype suggests the existence of a very complex variability of this syndrome, possibly originated by the interaction of genetic background and environmental factors [2,44,45]. However, it has been proposed that additional simultaneous mutation may contribute to phenotype variation [46].

The developmental defects associated with NBCCS are mostly due to haploinsufficiency. Instead, as reported for other tumor suppressor genes, tumors develop in the presence of two genetic alterations. The first genetic defect is inheritance of a mutation and the second hit is inactivation of the normal homologue by environmental mutagenesis or random genetic rearrangement that definitely confer ligand-independent activation of the Hh pathway, cellular growth, genetic instability and, potentially, tumor development [47]. This loss has also been observed in basocellular carcinomas independently of the syndrome, in the meduloblastoma, in odontogenic keratocysts and trichoblastomas [6,48]. In sporadic BCCs, mutations of one homologue have been described at high frequency (67%) [49] and loss of heterozygosity (LOH) by nondisjunction, deletion, or mitotic recombination in the chromosomal region surrounding *PATCH1* gene has also frequently been found (93%) [50,51]. Interestingly, <50% of the *PTCH1* mutations in sporadic BCCs have the typical UVB signature [52]. Sporadic BCCs, which do not have mutations in this gene, carry gain-of-function mutations in the Smoothened (*SMO*) gene, another member of the Hh pathway [53,54]. Thus, inactivation of *PTCH1* or oncogenic activation of *SMO* occurs in almost all BCCs. Therefore, deregulation of Hh signaling is considered the pivotal aberration in all BCCs (whether in syndromic and non-syndromic patients). Patched may function as a “gatekeeper gene” for BCC formation; i.e., a precursor cell gains a survival advantage with inactivation of a gatekeeper gene, and inactivation would be necessary before clonal expansion and accumulation of multiple genetic events preceding BCC formation [55].

### 1.3. Hedgehog Signaling

**Figure 1:**
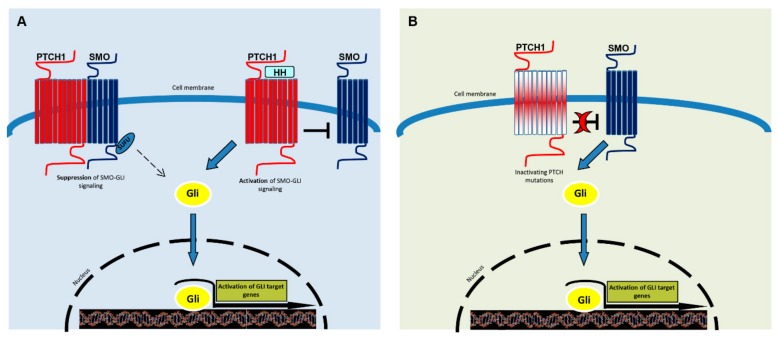
A simplified representation of the Hedgehog signaling pathway. SMO is the key signal transducer of the Hh pathway. (**A**) In the absence of Hedgehog (Shh, Dhh, Ihh) ligands, PTCH1 inhibits SMO signaling. Gli molecules are processed with the help of SuFu molecules into repressor forms, which disable the Hh signaling pathway. The binding of Hh promotes SMO conformational change, leading to activation of the GLI transcription factors (the activators Gli1 and -2 and the repressor Gli3). Activated GLI accumulates in the nucleus and controls the transcription of hedgehog target genes. (**B**) Mutated PTCH1 does not inhibit SMO and the pathway results activated, as in the presence of ligands.

The Hh pathway is one of several major signaling pathways that demonstrated a connection between embryogenesis, regeneration, and cancer, highlighting common signaling networks in these processes [12,36]. Hh signaling also participates in control of adult stem cell proliferation, tissue homeostasis maintenance, and regeneration [56,57]. Hh signaling is turned on locally as a physiological response to wound, ischemic stroke, or myocardial infarction [58,59]. The hedgehog pathway appears to be evolutionary conserved from Drosophila to vertebrates [60,61]. This raises the important question of how long-term use of Hh inhibitors can disturb normal physiology. Despite overall similarity, many vertebrate-specific components have been identified [62]. In contrast to most signaling pathways, in which the ligand activates its receptor, in Hh signaling, the ligand inhibits the PTCH receptor. In the canonical pathway, binding of Sonic Hedgehog (SHH), the most widely expressed hedgehog protein to its receptor, PTCH, releases inhibition on SMO, which transduces the signal to the cytoplasm [63,64,65,66], ultimately leading to the activation of the glioma-associated oncogene homolog zinc finger transcription factors (Gli1, -2 and -3) [67,68,69] (Figure 1). Among them, Gli1 exists only as its activated form, while Gli2 and Gli3 have both activator and repressor forms. Gli1 and Gli2 increase the expression of key regulators of G1/S and G2/M phase progression of the cell cycle, thereby promoting the transition from a quiescent to the proliferative state. The pathway normally is regulated by the spatially and temporally restricted expression of Shh, and two other less expressed homologues, Desert hedgehog (DHH), and Indian hedgehog (IHH). In the absence of functional PTCH, smoothened may be constitutively active, independent of hedgehog control, and may induce overexpression of several target genes including Wnts, CyclinD1 and D2, N-myc, Snail, the anti-apoptotic factor Bcl2 as well as feedback genes of Hh signaling (PATCH1 and 2, Gli1 and huntingtin interacting protein-1, Hip1) [70,71]. The Hh signaling pathway is in conjunction with other important pathways, such as epidermal growth factors (EGF/EGFR), transforming growth factor-β (TGF-β), and Wnt/β-catenin signaling [72,73]. Gli1 is also implicated in replicative immortality achieved by human telomerase reverse transcription transcriptase (hTERT) protein expression regulation [74].

## 2. Hh Signaling in Tumor Microenvironment

In addition to cancer cells, tumors exhibit a co-operational and evolving coexistence of a number of cell types comprehending immune cells, endothelial cells, pericytes, adipocytes, and fibroblasts. These cells, of which fibroblasts constitute a major part, are embedded in the extracellular matrix composed of a multitude of scaffold proteins (polysaccharides, fibrous proteins, and proteoglycans), growth factors, chemokines and cytokines, that are released by various cell types. These autocrine/paracrine acting factors have a crucial role in the survival and progression of tumors [75]. Evidence has been documented that alteration in the microenvironment plays an important role in both BCC and squamous cell carcinoma (SCC) [76,77,78]. In the skin, accumulation of senescent fibroblasts mostly due to photo-aging alters the surrounding tissue into a neoplasia-promoting environment [78]. BCC-associated CAF expression profile is mainly linked to extracellular matrix metabolism such as MMPs, metallopeptidase, lysol oxidases, 4-hydroxylase, fibronectin, fibronectin, proteoglycans and factor involved in epithelial to mesenchymal transition [78]. Moreover, in tissue surrounding BCC abundant collagen XI, which is not normally present in skin, argues for a pro-invasive phenotype. BCC peritumoral stroma also is a source of cytokines and chemokines implicated in local immunosuppression. In BCC lesion, CAF expressed an increased level of two WNT pathway proteins, WISP1 and Lef1 [78]. Although several reports have described a cell-autonomous autocrine role for Hh signaling in tumors, recent data support an additional paracrine model of Hh-mediated tumorigenesis, in which neoplastic cells secrete Hh ligand to activate tumor-promoting hedgehog target genes in adjacent stroma [79,80]. Even though in NBCCS patients the activation of the Hh pathway originates in a different context, specifically by an intrinsic ligand-independent mechanism, the resulting stroma may support cancer development similarly to tumor stroma. Hence, NBCCS represent a useful model not only to evaluate keratinocytes during BCC onset but also for studying BCC/microenvironment molecular cross-talk.

### 2.1. Hh Signaling in Cancer-Associated Fibroblasts

Previous studies on various human cancers suggested that Hh signaling effects tumourigenesis by controlling cell proliferation, angiogenesis, and epithelial-mesenchymal transition (EMT) in an autocrine–juxtacrine manner [81]. However, tumor microenvironment may significantly influence cancer evolution, both in cancer cells hedgehog-dependent and -independent [82]. In the process of tumor formation, quiescent fibroblasts change to adapted reactive stromal elements phenotypically closely resemble myofibroblasts with enlarged spindle-shape and elongated projections, expressing α-smooth muscle actin. Collectively, the desmoplastic reaction secondary to neoplasm generates scar tissue similar to the wound healing process [83,84]. Thus, tumors are often referred to as “wounds that never heal” [82]. Cancer-associated fibroblasts (CAF) express specific markers such as fibroblast activation protein (FAP), fibroblast-specific protein 1 (FSP1), neuronglial antigen-2 (NG2), vimentin, tenascin (TNC), and display an increased secretion of various extracellular matrix (ECM) proteins (i.e., collagens I, III, IV), proteoglycans (i.e., fibronectin, laminin), chemokines (e.g., CXCL and CCL), cytokines and other tumor-promoting factors which affect vascularisation (i.e., platelet-derived growth factor (PDGF), vascular endothelial growth factor (VEGF)), stromal-derived factor-1 (SDF-1), matrix metalloproteinase (MMPs), and tissue inhibitors of MMPs (TIMPs), capable of promoting tumor cell invasiveness and survival. Intensive tumor-stroma bidirectional cross-talk is responsible for fibroblast secretome switch onto CAF secretome [85,86,87]. Cancer stimulates stromal cells to also produce a huge number of growth factors that support neoplastic cell proliferation (i.e., TGF-β, epidermal growth factor (EGF), hepatocyte growth factor (HGF) or fibroblasts growth factor (FGF), and Wnts [88,89]. Recent studies have shown that Hh signaling activation, driven by ligand expression in carcinoma cells, can modulate the stromal cells to create a favorable environment for faster cancer progression [90,91].

In mice models, increased Hh activity in tumor fibroblasts results in overexpression of VEGF which increases endothelial cell proliferation and vascular density in tumor [92]. Furthermore, Hh signaling exerts a critical role in tumor-associated macrophages (TAMs) immunosuppression. In myeloid cells, sonic hedgehog secreted by tumor cells induces functional M2 polarization with consequent suppression of CD8 + T cell recruitment via reduction of CXCL9 and CXCL10 [93,94]. Wei and coauthors recently demonstrated that SHH ligands increase PTCH1 and Gli1 expression leading to Hh signaling pathway activation in human CAF but not in normal fibroblasts. In the same study, Hh signaling stimulation augmented CAF proliferation and confirmed lymphangiogenesis in a xenograft model [95]. The Hh pathway also supports tumor cell invasion by stimulating cell motility and MMPs expression and by directly targeting genes implicated in EMT. In addition to paracrine factors, a tumor-stromal direct contact seems to be necessary for Hh signaling-dependent mobilization of non-small cell lung cancer cells [96]. One important point is the fact that in in situ skin tumor, cancer cells and stromal components can communicate through the basement membrane by diffusible factors while exclusively in invasive tumor, cancer and stromal cells are in direct contact and establish a complex cross-talk capable of altering fibroblast phenotype. Therefore, alterations in the composition of basement membrane represents an early microenviroment modification for skin cancer progression. In general, indirect CAF-cancer cell interaction is responsible for tumor growth promotion by the paracrine release of pro-mitogenic factors, whilst direct CAF-cancer contact mediates cell migration and invasion [88]. NBCCS fibroblasts, at least during BCC initiation, are in a situation similar to those of in situ non-invasive BCC, whereas CAF are more likely in proximity or in direct cell–cell contact with tumor cells. This unique scenario does not find similarity in non-syndromic skin carcinoma and could, in part, explain the prevalent indolent phenotype of NBCCS-BCCs.

Moreover, the Hh pathway supports cancer genomic instability and inflammation [94]. Another important issue regarding the role of fibroblasts in cancer evolution is the frequency of somatic genetic mutations in CAFs. Manifold studies demonstrated that high percentage of CAFs undergo genetic alterations, including loss of heterozygosity or mutation of tumor suppressor genes (i.e., PTEN and p53) [97]. This is a relevant point for cutaneous tumor since skin is constantly exposed to extrinsic stressor including ultraviolet radiation. Until now, somatic mutations in the Hh pathway have not been investigated in sporadic skin cancer.

### 2.2. Dermal Fibroblasts from Gorlin Syndrome Have Phenotypic Traits Reminiscent BCC Cancer Associated Fibroblasts

The first evidence that NBCCS fibroblasts are prone to acquire CAF phenotype arose from a report by Majmudar and co-workers in 1993. In this study, all the fibroblast cell cultures isolated from skin adjacent to BCC tumors of NBCCS patients demonstrated overexpression of MMP3, whereas in corresponding nonsyndromic fibroblast cultures, abnormal MMP3 expression was restricted to 25% of BCCs. More interestingly, the abnormal expression of MMP3 has been found extended to uninvolved skin fibroblasts, demonstrating the CAF-prone phenotype in GS patients [98]. Next, this observation was extended to other CAF markers, spanning from molecules involved in tissue organization such as MMP1, collagen type 11 alpha 1 (COL11A1), matrix gla protein (MGP), and TNC to chemokines as CXCL12, and other bioactive factors governing cell proliferation and survival as keratinocyte growth factors (KGF), angiopoietin-related protein 2 and 4 (ANGPTL2/4). Among the Wnt pathway regulator factors, secreted frizzled-related protein-1 and 2 (SFRP2/2), Dickkopf 1 (DKK1), and WNT inhibitory factor 1 (WIF1), WNT1, inducible signaling pathway protein 2 (WISP2) have been found upregulated, while DKK3 was downregulated. Activation of the WNT/β-catenin signaling pathway has been increasingly appreciated in CAF biology [99,100] especially in the skin background [101]. The expression level of proteins involved in extracellular matrix remodeling, such as MMP1, MMP3, TNC as well as KGF and stroma cell-derived factor 1 alpha, has been associated with the NBCCS-BCCs aggressive clinical phenotype since patients presenting a high number of large BCCs show a higher level of expression compared to non-aggressive clinical phenotype [102]. By contrast, the difference in the protein profile among distinct genetic groups (missense and nonsense) failed to demonstrated definitive data [103,104]. The idea that fibroblasts carrying heterozygous *PTCH1* mutations present a phenotype overlapping with those of skin CAF is also supported by the adhesion properties and intracellular cytoskeleton organization since α-SMA and vinculin are highly expressed and organized than in healthy fibroblasts [104]. Functional dermo-epidermal interaction in 3D organic cultures evidenced reduced epidermal thickness and dysregulation of keratinocyte differentiation proteins when healthy keratinocytes were maintained in the presence of NBCCS fibroblasts [105]. In the same experimental system, diffusible factors produced by NBCCS fibroblasts strongly stimulate the expression of p53 in an organotypic in vitro model containing normal keratinocytes, whereas carcinoma cells carrying mutate p53 acquired an invasive phenotype in the presence of NBCCS dermis [106]. The possible contribute of syndromic dermal cells in photodynamic therapy success has also been highlighted demonstrating that GS fibroblasts are predisposed to accumulate UV-dependent oxidative stress and consequence cell death [106]. In addition to an abnormal response to UV, NBCCS fibroblasts display increased oxidative perturbation following radiation associated with a marked deficiency in aldehyde dehydrogenase 1A1, one of the rate-limiting enzymes in retinoic acid synthesis [105]. Recently, additional evidence arose from microRNAs profiling in fibroblasts derived from Gorlin syndrome patients. Two miRNAs, has-miR-196a-5p and has-miR-4485, involved in mitochondrial function and tumorigenesis [107], were found to be downregulated and upregulated, respectively, [108] prospecting innovative miRNA-based therapies.

Even if the overall description of NBCCS fibroblasts in the literature strongly argues for a cancer associated phenotype, it is important to take into consideration that CAFs are reported to be a heterogeneous cell population that include both tumor-promoting and tumor-suppressing phenotypes expressing partially overlapping markers [68]. The provocative possible involvement of syndromic fibroblasts in the indolent phenotype presented by NBCCS-BCCs has not been investigated yet. Moreover, CAFs progressively co-evolve with the disease, whereas NBCCS fibroblasts are genetically determined to assume cancer microenvironment features. Co-evolution of CAFs and neoplastic cells includes intrinsic and extrinsic aging since sporadic BCCs are particularly prevalent in the elderly. Senescent phenotype is not necessary involved in NBCCS predisposition to BCC since hyperproliferation frequently develops early in life and in sun-unexposed body area. Lastly, in tumor, the stroma contains activated fibroblasts that are also increased in number, while no alteration in the number of this type of cells has been reported in NBCCS skin. The insight of all these interesting points needs to be clarified in future investigations.

## 3. Conclusions

Although many aspects of the *Drosophila* Hh pathway are conserved in vertebrates, mechanistic differences between the two species have begun to emerge, disabling some important notions previously acquired. Thus, patients with GS provide an important human model of hyperactivity in hedgehog signaling. On the other hand, the similarity of syndromic fibroblasts to CAFs complemented our knowledge about the predisposition of these patients to develop a huge number of BCCs in early life and in sun-unexposed body areas. In line with the idea that fibroblasts may be key players in cancer-prone hereditary diseases, abnormal dermal fibroblast activation and extracellular matrix remodeling has been described as a common hallmark of genodermatoses (recessive dystrophic epidermolysis bullosa, Kindler syndrome and xeroderma pigmentosum) [109]. It is incontrovertible that the accumulation of senescent cells in the skin provides a better milieu and stromal support for tumor cells. According to this notion, it will be of great importance to understand if NBCCS genetic defects are linked to the accumulation of senescent fibroblasts and limited regenerative potential independent of sun explosion and chronological age. Future research will clarify whether regenerative therapies are useful for Gorlin syndrome patients. Moreover, the characterization of NBCCS-derived fibroblasts and correlation of molecular data with clinical data may help to address the question of their role in therapy efficacy and resistance as well as provide a useful model to evaluate new therapeutic approaches of BCC. In particular, the effect of promising treatment, such as target therapies on the NBCCS dermal microenvironment, needs to be investigated.

## Abbreviations

NBCCS	Nevoid basal cell carcinoma syndrome
BCC	Basal cell carcinoma
PTCH	protein patched homolog
Hh	Hedgehog
CAF	Carcinoma associated fibroblast
iPCS	induced pluripotent stem cells
UV	ultraviolet
UVB	ultraviolet B
SUFU	suppressor of fused homolog
LOH	loss of heterozygosity
SMO	Smoothened gene
SHH	Sonic Hedgehog
Gli1, 2, 3	glioma-associated oncogene 1,2,3
DHH	Desert hedgehog
IHH	Indian hedgehog
Wnt	wingless signaling pathway
N-myc	neuroblastoma oncogene
Bcl2	B-cell lymphoma 2
Hip1	Huntingtin interacting protein-1
EGF	epidermal growth factor
EGFR	epidermal growth factor receptor
SCC	Squamous cell carcinoma
TGF-β	transforming growth factor beta
hTert	human telomerase reverse transcription transcriptase
EMT	epithelial-mesenchymal transition
FAP	fibroblast activation protein
FSP1	fibroblast-specific protein 1
GS	Gorlyn Syndrome
NG2	neuroglial antigen-2
TNC	tenascin
ECM	extracellular matrix
CXCL	chemokines
CCL	cytokines
PDGF	platelet- derived growth factor
VEGF	vascular endothelial growth factor
SDF-1	stromal derived factor-1
MMP	matrix metalloproteinase
TIMP	tissue inhibitors of MMP
HGF	hepatocyte growth factor
FGF	fibroblasts growth factor
TAMS	tumor-associated macrophages
CXCL9	chemokine ligand 9
CXCL10	chemokine ligand 10
PTEN	phosphatase and tensin homolog
COLL11A1	collagen type 11 α1
MGP	Matrix gla protein
TNC	tenascin
KGF	keratinocytes growth factor
Angptl2/4	angiopoietin-related protein 2 and 4
SFRP	secreted frizzled-related protein
DKK1	Dickkopf1
WIF1	Wnt inhibitory factor 1
WISP2	Wnt1-inducible-signaling pathway protein 2
Wnt1	wingless-type MMTV integration site family, member 1
α-Sma	*α smooth muscle actin*
miRNA	small non-coding RNA molecule
